# Brain Structure Links Loneliness to Social Perception

**DOI:** 10.1016/j.cub.2012.08.045

**Published:** 2012-10-23

**Authors:** Ryota Kanai, Bahador Bahrami, Brad Duchaine, Agnieszka Janik, Michael J. Banissy, Geraint Rees

**Affiliations:** 1 UCL Institute of Cognitive Neuroscience, 17 Queen Square, London WC1N 3AR, UK; 2 Wellcome Trust Centre for Neuroimaging, Institute of Neurology, University College London, 12 Queen Square, London WC1N 3BG, UK; 3 Interacting Minds Project, Institute of Anthropology, Archaeology, Linguistics, Aarhus University, and Centre of Functionally Integrative Neuroscience, Aarhus University Hospital, Norrebrogade 44, Building 10 G, 8000 Aarhus C, Denmark; 4 Department of Psychological and Brain Sciences, Dartmouth College, Moore Hall 6207, Hanover, NH 03755, USA; 5 Department of Psychology, Goldsmiths, University of London, New Cross, London SE14 6NW, UK

## Abstract

Loneliness is the distressing feeling associated with the perceived absence of satisfying social relationships [1]. Loneliness is increasingly prevalent in modern societies [2, 3] and has detrimental effects on health and happiness [4, 5]. Although situational threats to social relationships can transiently induce the emotion of loneliness, susceptibility to loneliness is a stable trait that varies across individuals [6–8] and is to some extent heritable [9–11]. However, little is known about the neural processes associated with loneliness (but see [12–14]). Here, we hypothesized that individual differences in loneliness might be reflected in the structure of the brain regions associated with social processes [15]. To test this hypothesis, we used voxel-based morphometry and showed that lonely individuals have less gray matter in the left posterior superior temporal sulcus (pSTS)—an area implicated in basic social perception. As this finding predicted, we further confirmed that loneliness was associated with difficulty in processing social cues. Although other sociopsychological factors such as social network size, anxiety, and empathy independently contributed to loneliness, only basic social perception skills mediated the association between the pSTS volume and loneliness. Taken together, our results suggest that basic social perceptual abilities play an important role in shaping an individual’s loneliness.

## Highlights

► Loneliness is correlated with the volume of posterior superior temporal sulcus (pSTS) ► The pSTS volume predicted the ability to recognize social signals ► Lonely individuals have difficulty in recognizing eye gaze direction ► Social network size, anxiety, and empathy also shape loneliness

## Results

### Experiment 1. Voxel-Based Morphometry of Loneliness

**Figure 1:**
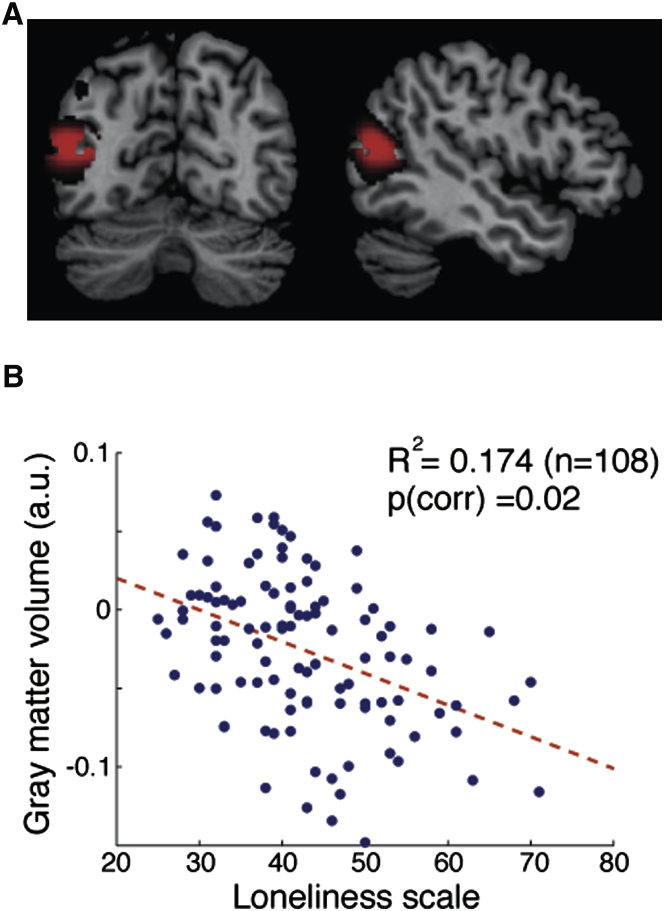
Gray Matter Volume Correlated with Loneliness Scale (A) The left STS in which variability in gray matter volume exhibited significant negative correlation with loneliness scale (n = 108) is superimposed on a standard T1-weighted template brain in MNI stereotactic space. The significant cluster is shown at t > 2.3 for visualization purpose. (B) A scatterplot between loneliness scale and pSTS volume adjusted for age, gender, and total gray matter volume is shown for illustration purpose only. Statistical inference was based on the p value corrected for multiple comparisons across the whole brain at a cluster level with nonstationary correction [44].

We correlated brain structure and reported loneliness in a sample of 108 healthy adults (see Experimental Procedures) and found a large significant cluster in the posterior superior temporal sulcus (pSTS) in which the regional gray matter volume negatively correlated with individual differences in loneliness (cluster size = 3,837 mm^3^, p[corr] < 0.05 nonstationary). Lonely individuals had smaller gray matter volume in the pSTS cluster (Figure 1). The peak voxel was situated within the middle temporal gyrus (T[103] = 4.66, Z = 4.42, R^2^ = 0.174, p[FWE-corr] = 0.02, MNI coordinate x = −48, y = −69, z = 15). We did not find any significant cluster that positively correlated with loneliness (p[corr] > 0.05 nonstationary correction; see Table S1 available online for uncorrected results).

Given the known functions of pSTS in social perception [16, 17], it appears unlikely that the volume of the left pSTS directly mediates subjective experiences of loneliness per se. The pSTS region is thought to be involved in initial stages of social perception combining different sensory cues such as eye gaze, hand action, and body movements [16]. In particular, the position of the pSTS cluster revealed in our VBM result is close to the locus where activations are elicited when viewing the eye gaze of others [18]. Specifically, our pSTS locus overlapped with the mean coordinate of the loci sensitive to eyes (x = −48, y = −55, z = 6; see [16] for a meta-analysis).

### Experiment 2. Social Perception and Loneliness

Taking our findings from experiment 1 with the previously established functional role of pSTS leads to the intriguing hypothesis that lonely individuals might have deficits in basic social perception.

**Figure 2:**
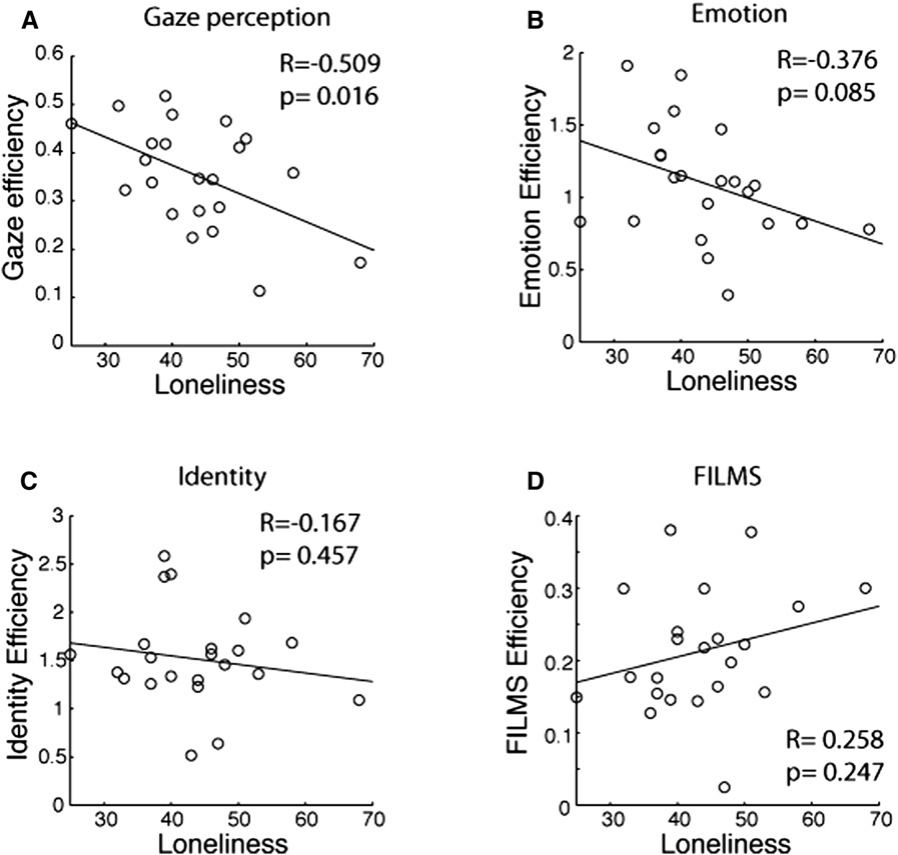
Relationship between Loneliness Scale and Performances for Social Perception Tasks (n = 22) Abnormal gaze detection task (A), same-different emotion discrimination task (B), same-different identity discrimination task (C), and films emotion recognition task (D). See Experimental Procedures for full details of the tasks.

**Figure 3:**
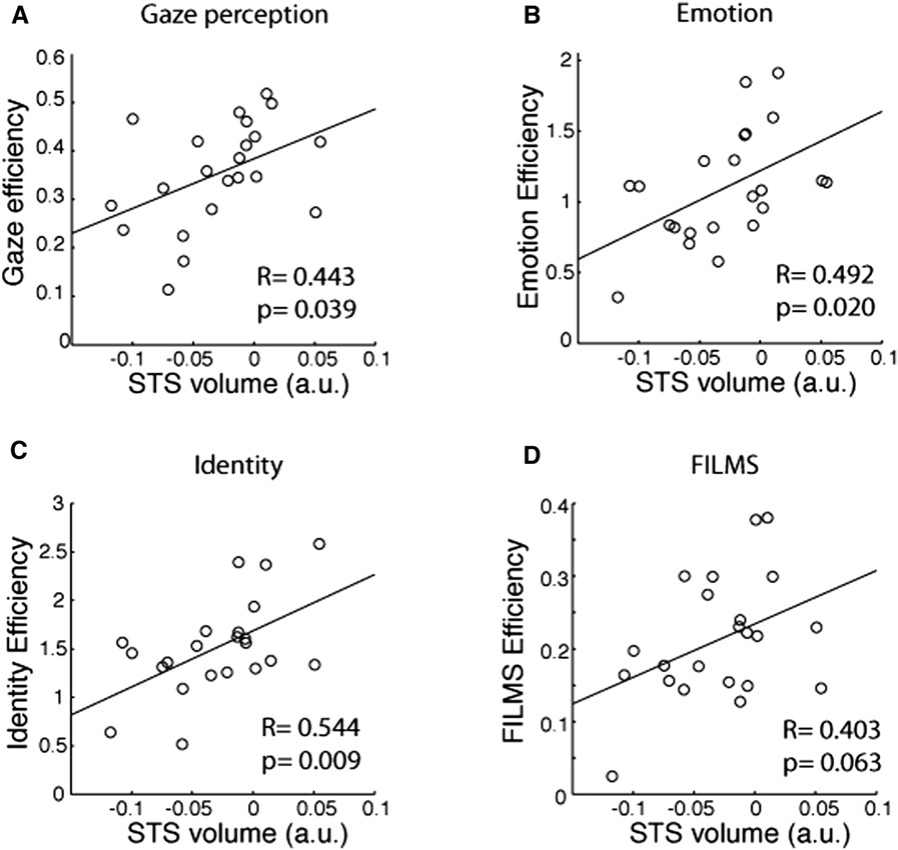
Relationship between Regional pSTS Volume and Behavioral Performances in Social Perception Tasks (n = 22) Abnormal gaze detection task (A), same-different emotion discrimination task (B), same-different identity discrimination task (C), and films emotion recognition task (D). See Experimental Procedures for full details of the tasks.

We tested this hypothesis in a subset of the original participants (n = 22) using a gaze perception task. Participants were shown three faces and asked to judge which face showed strabismic gaze (eyes not aligned properly). We found that the ability to process eye gaze information was negatively correlated with self-reported loneliness (Figure 2A; R = −0.51, T[20] = −2.64, p = 0.015). We replicated this association in an independent sample (n = 38) using a more naturalistic gaze task and confirmed the specificity of this association, because it was not observed for a nonsocial face perception task (see Experiment S1 and Figure S1). Moreover, the efficiency of eye gaze processing was significantly correlated with the regional gray matter volume of the pSTS cluster (Figure 3A; R = 0.441, T[20] = 2.21, p = 0.038).

Although the results so far suggest that pSTS, eye gaze processing, and loneliness are linked with one another, whether the gaze perception ability mediates the relationship between the volume of pSTS and loneliness remained uncertain. We therefore computed the partial correlation between pSTS volume and loneliness while regressing out the contribution of eye gaze performance. This revealed that the original significant correlation between pSTS gray matter volume and loneliness in this sample (R = −0.454, p = 0.03) vanished after controlling for individual differences in eye gaze performance (R = −0.138, p = 0.550). This supports the notion that the negative correlation between pSTS volume and loneliness was mediated by the efficiency of perceiving eye gaze.

To examine whether the relationship between loneliness and social perception via pSTS was specific to eye gaze perception, we examined participants’ abilities on several other types of social perception tasks, namely, facial expression discrimination [19, 20], facial identity discrimination [19, 20], and facial emotion recognition [20, 21]. The correlation between loneliness and the ability to discriminate facial emotional expressions did not reach statistical significance (Figure 2B; R = −0.376, T[20] = −1.81, p = 0.085). However, this performance was significantly correlated with the gray matter volume in the pSTS cluster (Figure 3B; R = 0.492, T[20] = 2.528, p = 0.020). Similarly, sensitivity to face identity did not correlate with loneliness (Figure 2C; R = −0.167, T[20] = −0.759, p = 0.457) but significantly correlated with the gray matter volume in the pSTS cluster (Figure 3C; R = 0.544, T[20] = 2.899, p = 0.009). Performance in the emotion recognition task (measured by efficiency score) did not significantly correlate with loneliness (Figure 2D; R = 0.258, T[20] = 1.19, p = 0.257) or the gray matter volume in the pSTS cluster (Figure 3D; R = 0.403, T[20] = 1.97, p = 0.063). Taken together, loneliness was correlated with the perception of eye gaze, whereas the gray matter volume of the left pSTS correlated more broadly with processing social cues from faces.

### Experiment 3. Social Network Size and Loneliness

Next, we examined whether social network size could explain the correlation between loneliness and pSTS. Previously, we have shown that the gray matter volume of the middle temporal gyrus (MTG) region abutting the pSTS region associated with loneliness correlates with online social network size [22]. Thus, it is possible that the link between pSTS and loneliness was mediated by individual differences in social network size. We therefore examined whether the pSTS-loneliness correlation could be explained by social network size by collecting data on social network size from a subset of the original participants (n = 45). We found that although social network size was strongly correlated with the loneliness scale (R = −0.617, T[43] = −5.144,p < 0.001), factoring out the social network scale did not affect the pSTS-loneliness correlation (R = −0.395, T[42] = −2.789, p = 0.008; original correlation in this sample, R = −0.386, T[42] = −2.743, p = 0.009). There was also no correlation between pSTS size and the social network scale (R = 0.125, T[43] = −0.823, p = 0.413), because the overlap between the pSTS cluster for loneliness the cluster previously reported for online social network size overlapped only less than 1% (see Figure S2). These results together indicate that the left pSTS gray matter volume and social network size independently predict an individual’s loneliness.

### Experiment 4. Trait Anxiety and Loneliness

Loneliness scores are correlated with other mood factors such as anxiety and depressive symptoms [23]. To examine whether such mood measures mediated the association between the volume of pSTS and loneliness, we administered the STAI inventory to collect data on trait anxiety from 61 of our original participants in experiment 1.

We replicated previous findings [23] showing that trait anxiety is highly correlated with loneliness score (R = 0.596, p < 0.001). We extracted the pSTS volume from MRI scans of this subpopulation of participants and found that trait anxiety also showed a tendency to negatively correlate with pSTS volume, but this did not reach statistical significance (R = −0.226, p = 0.08). Because this subpopulation was selected from the participants in experiment 1, we observed the expected negative correlation between loneliness score and pSTS volume in this subsample (R = −0.596, p < 0.001).

To examine whether this correlation was (partially) mediated by trait anxiety, we tested whether the correlation was weakened by inclusion of anxiety as a covariate. However, the loneliness-pSTS correlation was unaffected by regressing out the contribution of anxiety (R = −0.568, p < 0.001). These results indicate that the volume of left pSTS and trait anxiety independently contribute to loneliness score.

### Experiment 5. Empathy and Loneliness

Finally, we examined whether loneliness was associated with aspects of empathy using the Interpersonal Reactivity Index (IRI), which measures fantasy scale (FS), perspective taking (PT), personal distress (PD), and empathic concern (EC) [24, 25]. We collected these IRI subscales from a subset (n = 95) of the participants studied in experiment 1. We found that the loneliness score significantly correlated with PD (T[93] = 3.59, R = 0.349, p < 0.01, Bonferroni corrected), but not with other subscales (FS, T[93] < 1, R = 0.028, p = 0.316; PT, T[93] = −1.97, R = −0.200, p = 0.208; EC, T[93] = −2.30, R = −0.233, p = 0.092, Bonferroni corrected). This is in line with previous reports that people with a high PD score show poor social perception and social competence [24] and further supports our findings that loneliness is related to reductions in social perception. None of these subscales were, however, significantly associated with the volume of the left pSTS (all p > 0.05).

## Discussion

Our experiments show that individual differences in the expressed trait of loneliness are linked with variations in the gray matter volume of left pSTS. This region has been implicated in several fundamental aspects of processing social information. However, it is unlikely that pSTS directly (or any other single brain area per se) mediates such a complex cognitive quality as the transient feeling of loneliness. This brain region is causally involved in perception of social stimuli such as biological motion [26, 27] and gaze direction [28], suggesting that the feeling of loneliness may be associated with deficits in these basic social perceptual skills. Our behavioral experiments confirmed this hypothesis by showing significant correlations between individual variability on the reported loneliness scale and objectively measured skills relevant to social perception such as the performance of eye gaze perception. Furthermore, the volume of pSTS also predicted such basic social skills, which confirms the relevance of the same pSTS region in those social perception tasks.

Our findings indicate that lonely individuals have deficits at a relatively early stage of processing social cues. Lonely individuals are low in social skills [29, 30] and have poor sensitivity to nonverbal communication [31], whereas they are proficient in verbal communication [32]. People with poor social skills are more likely to become lonely when they encounter negative stressful life events [33]. This finding is in line with the hypothesis that social skills deficits are antecedents of loneliness [34]. However, it should be noted that in those studies, social skills were measured by different methods such as questionnaires (e.g., the Social Skills Inventory, [35]) and it remains unclear whether social skills measured by questionnaire items (e.g., “At parties I can instantly tell when someone is interested in me.”) correspond to basic aspects of social perception measured in the laboratory as here.

Subjective loneliness modulates brain activations to pleasant and unpleasant pictures of other people [14]. Specifically, lonely individuals show weaker activation in the ventral striatum when viewing pictures of pleasant social events than when viewing pleasant pictures of nonsocial objects, whereas this pattern is reversed for nonlonely individuals. Furthermore, lonely individuals show weaker activations in bilateral temporoparietal junction (TPJ) to unpleasant social pictures of people compared to unpleasant pictures of objects. Although functional and structural correlates of loneliness may be regionally dissociated, both the previous functional study [14] and our current study point to the idea that loneliness is reflected in the way the brain processes visually presented social cues. In future research, conjoint measurements of structure and function will be needed to establish their relationships.

The correlation we observed between pSTS and loneliness seems to be specifically mediated by the ability to process eye gaze information, because factoring out the eye gaze performance abolished the correlation between pSTS and loneliness. This was not observed with other measures such as social network size, anxiety, and empathy (personal distress in particular) that were correlated with loneliness. These results suggest that a multitude of social and psychological factors contribute to loneliness score, but their association with loneliness is independent of the pSTS structure. We speculate that interindividual differences in those factors may also have a basis in the structure of other brain regions [22, 25, 36–39], but their associations with loneliness may have been too weak to be detected within our current sample size (n = 108). Further investigation with a larger sample may help reveal more regions that are relevant for individual differences in loneliness. Future studies may benefit from asking participants under what kind of circumstances they feel lonely, because such qualitative data may help us understand how multiple pathways lead to loneliness in different individuals.

Because of the cross-sectional nature of our present study, we cannot determine the direction of causation between loneliness, social perceptual abilities, and the volume of the left pSTS. One appealing possibility is that poor ability to recognize social cues may lead to social isolation and loneliness. For example, people who are poor at reading social cues may experience difficulty in developing social relationships. This hypothesis predicts that improvements in social perception by training may increase the quality and quantity of social interaction and thereby mitigate the degree of subjective loneliness. A recent meta-analysis of intervention studies that aimed to reduce loneliness with various strategies showed that the most effective intervention for treating loneliness is improving maladaptive social cognition [40]. Thus, it seems worth considering provision of training on basic social perception skills such as detecting eye gaze direction as an intervention to reduce loneliness. Conversely, it is also possible that socially isolated individuals have less frequent social contacts and therefore have less opportunity to develop sensitivity to social visual cues. This hypothesis predicts that social environment changes (e.g., freshmen who leave family and friends behind or people who start living alone) that make people lonely [41] would impact on basic social perception skills. Longitudinal or intervention studies will be particularly useful to disentangle complex relationships between loneliness, social perception skills, and relevant brain areas.

## Experimental Procedures

### Experiment 1. Voxel-Based Morphometry of Loneliness

#### Participants

A total of 108 healthy volunteers with normal or corrected to normal vision (aged 18–32, mean 23.5 ± 4.37 SD, 62 female) were recruited from the University College London subject pool. The experiments were approved by the local ethics committee, and participants gave written informed consent.

#### Assessment of Loneliness

All participants were asked to fill out the UCLA Loneliness Scale Questionnaire [8].

#### MRI Data Acquisition and Analysis

MR images were acquired on a 1.5-T Siemens Sonata MRI scanner (Siemens Medical, Erlangen, Germany) using a T1- weighted 3D Modified Driven Equilibrium Fourier Transform (MDEFT) sequence. A multiple regression analysis was performed on coregistered gray matter images [42, 43] preprocessed in SPM8 to determine regions in which gray matter density showed a correlation with the UCLA Loneliness Scale [8]. The age, gender, and total gray matter volume of individual brains were included in the design matrix as covariates of no interest. We used a threshold of p(corr) < 0.05 corrected for multiple comparisons at a cluster level using nonstationary correction [44].

### Experiment 2. Social Perception and Loneliness

We contacted the participants in experiment 1 and asked them to take part in follow-up experiments. A total of 22 healthy volunteers (aged 19–30, mean 22.7 ± SD 3.9, 15 females) were tested on four social perception tasks: abnormal gaze detection task, emotional expression discrimination task, identity discrimination task, and films emotion recognition task (see Supplemental Experimental Procedures for full details).

### Experiment 3. Social Network Size and Loneliness

Forty-five participants recruited from the UCL student community (aged 18–30 mean 23.2 ± SD 3.6, 52 females) completed the social network size questionnaire [45]. A normalized social network size was computed for each participant by averaging the Z scores for all the questions items (see Supplemental Experimental Procedures).

### Experiment 4. Anxiety and Loneliness

Sixty-one participants (aged 18–39, mean 23.5 ± SD 4.5, 43 females) from the population studied in experiment 1 completed the STAI for trait anxiety consisting of 20 question items (Form Y) [46].

### Experiment 5. Empathy and Loneliness

Ninety-five participants (aged 18–39, mean 22.3 ± SD 4.3, 53 females) from the population studied in experiment 1 completed the Interpersonal Reactivity Index (IRI) questionnaire consisting of 28 question items [24]. There were four subscales: fantasy scale (FS), perspective taking (PT), personal distress (PD), and empathic concern (EC) (see [24] for full details of the questionnaire).

## Supplemental Information

Document S1. Figures S1 and S2, Table S1, Experiments S1 and S2, and Supplemental Experimental Procedures
