# Supplementary material for: Brain Structure Links Loneliness to Social Perception
Source: Curr Biol. 2012 Oct 23;22(20):1975–9. doi: 10.1016/j.cub.2012.08.045 (PMC3510434; doi:10.1016/j.cub.2012.08.045)
Supplement: Document S1. Figures S1 and S2, Table S1, Experiments S1 and S2, and Supplemental Experimental Procedures [file mmc1.pdf]

Current Biology, Volume *22*

Supplemental Information

Brain Structure Links Loneliness to Social Perception

Ryota Kanai, Bahador Bahrami, Brad Duchaine, Agnieszka Janik, Michael J. Banissy, and Geraint Rees

Supplemental Inventory

1. Supplemental Figures and Tables

Figure S1, related to Figure 2

Figure S2, related to Figure 1

Table S1

Experiment S1

Experiment S2

2. Supplemental Experimental Procedures

3. Supplemental References

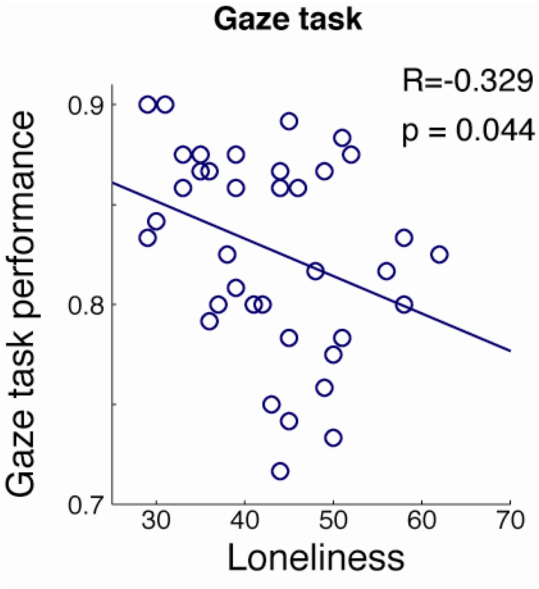

Figure S1. Replication of Negative Relationship between Loneliness Scale and Eye Gaze Discrimination Ability (n=38) with a Simple Gaze Direction Discrimination Task, Related to Figure 2

See Experimental Procedures for full details of the tasks.

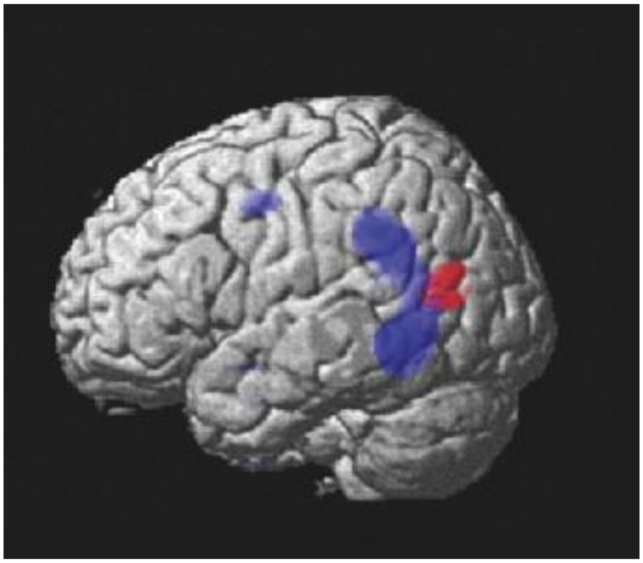

Figure S2. Comparison of the Loneliness Cluster in the pSTS (red) with the MTG Cluster Identified in our Previous Study (Ref. S7) on the Neural Correlates of Online Social Network Size (blue), Related to Figure 1

The MTG cluster is the lower half of the blue cluster, whereas the superior half corresponded to the temporoparietal junction (TPJ). However, the TPJ cluster did not reach statistical significance in our previous study. Note that the pSTS cluster reported in the current study did not overlap with MTG. The loneliness cluster was situated between the MTG and TPJ clusters and was slightly posterior to those clusters. The clusters are shown at a threshold of $p < 0.001$ uncorrected, both for the loneliness and Facebook studies for illustration purposes. The overlap of the two clusters was less than 1%.

Table S1. Summary of the Grey Matter Volume Associations with Loneliness

Area	H	MNI coordinates of peak voxel			Correlation (Pearson's r)	t(103)	Cluster size (mm^3)	Corrected P
		X	Y	Z				
Positive Correlation								
Middle temporal lobe	L	-70	-36	-9	0.33	3.55	682	0.306 n.s.
Inferior temporal lobe	L	-50	-7	-35	0.32	3.40	1640	0.217 n.s.
Heschl gyrus	L	-50	-13	7	0.33	3.53	506	0.251 n.s.
Fusiform gyrus	L	-32	-28	-21	0.32	3.46	395	0.247 n.s.
Insula	L	-34	-15	18	0.31	3.37	155	0.386 n.s.
Supramarginal gyrus	R	62	-24	31	0.31	3.26	216	0.330 n.s.
Negative Correlation								
Posterior STS	L	-48	-69	15	-0.42	4.66	3837	<0.05 *

Note: Corrected p values are the p values corrected for multiple comparisons at the cluster level across the volume of interest (see Experimental Procedures for details). Significant results are denoted by * ($p < 0.05$, corrected); n.s. indicates non-significant results; H = hemisphere; L = left; R = right.

Experiment S1. Replication of the Relationship between Eye Gaze and Loneliness

We tested whether the observed relationship between gaze perception and loneliness generalized to a more naturalistic gaze perception task and independent sample of participants. We asked 38 healthy new participants from whom loneliness scores were obtained to discriminate the direction of eye gaze of a person (left, right or straight ahead) displayed at fixation. Lonely individuals were again more likely to be poor at discriminating the direction of eye gaze (Fig. S1; $R=-0.329$, $T(36)=2.09$, $p=0.044$). In a control experiment, we examined whether the association between loneliness and behavioural performance was specific to eye gaze perception rather than more generic speeded decision making performance. To test this possibility, we administered a control task to the same 38 participants in which they were asked to simply indicate the position of a face image presented on the left, right or centre of the screen. We did not find significant correlation between loneliness score and the performance in this control task ($R=0.01$, $T(36)=0.03$, $p = 0.98$). Together with the results of Experiment 2, these results establish the specificity of the relationship between loneliness and eye gaze perception in two independent experiments.

Participants

We recruited new participants who did not participate in Experiment 1. We tested a total of 38 healthy volunteers with normal or corrected to normal vision (aged 18-35, mean 22.1 ± SD 3.6, 26 females). The experiments were approved by the local ethics committee and participants gave written informed consent.

Eye Gaze Perception Task

The gaze perception task was administered to investigate participants' abilities to perceive the gaze direction of another person. During the task, participants were shown a face presented centrally on the screen and were asked to indicate what direction the person was looking (either left, right or straight ahead) using a key press. On each trial, participants were shown a fixation cross (1000ms), followed by a face that remained on screen until participants responded. There were one hundred and twenty trials, comprised of forty trials in which the actor looked to the left, forty where the actor looked to the right, and forty where the actor looked straight ahead. These trials were split into three blocks of forty trials and were randomized within blocks. The stimuli were a set of computer generated Caucasian faces developed using FaceGen software (www.facegen.com). Five male and five female faces were used. The direction of gaze was manipulated for each face in order to generate nine levels of gaze direction (0%; 20% left/right; 40% left/right; 60% left/right; 80% left/right). Participant responses were recorded from the onset of the stimuli. Results reported for Experiment 2 remained significant even after controlling for the demographic variables of age and sex.

Eye Gaze Perception Control Task

The same 38 volunteers as the main experiment participated in the control task. The control task was administered to ensure that performance in the gaze perception task was specific to gaze and not response biases. In this task, the same stimuli from the gaze perception task were used, but appeared on the left, right or centre of the screen. Participants were asked to indicate where on the screen the image appeared. As with the gaze perception task, there were 120 trials. On 40 trials the image appeared to the left, 40 appeared to the right, and 40 appeared centrally. These trials were split into three blocks of 40 trials and were randomized within blocks. Participant responses were recorded from the onset of the stimuli.

Experiment S2. Autistic Traits and Loneliness

The results of the eye gaze experiment (Experiment 2) suggest that lonely individuals have similar perceptual deficits as those of autistic individuals. Autistic individuals frequently have difficulty with basic social perceptual skills such as eye gaze processing [S1-S3]. Interestingly, despite the common belief that autistic children prefer a solitary environment, high-functioning autistic children in fact express stronger feelings of loneliness than non-autistic controls [S4,S5]. This suggests that both in autistic and non-autistic individuals, part of the cause for strong feelings of loneliness might be poorer social perception. Indeed, the Autism-Spectrum Quotient questionnaire [S6].

We therefore tested whether there was any association between loneliness and autistic traits. Autistic traits for adults with average intelligence have been quantified by the Autism-Spectrum Quotient questionnaire [S6], which covers five domains associated with the autism spectrum: social skills, attention to detail, attention switching, communication skills and imagination. We collected AQ scores from a subset (n=48) of the participants studied in Experiment 1 above. We found a strong correlation between loneliness and total AQ score ($T(46) = 2.92$, $R = 0.3955$, $p < 0.05$, Bonferroni correction across the six comparisons). Further correlation analysis for each of the five subscales in AQ revealed that loneliness was strongly associated with the scales of social skills ($T(46)=5.15$, $R =0.605$, $p < 0.001$, Bonferroni correction), but not with any other subscales (all other p-values > 0.05). The specificity of the association between social skills and AQ scores corroborates the view that loneliness is associated with poor social skills.

Autism-Spectrum Quotient Questionnaire

Forty-eight participants (aged 18-33, mean 23.4 ± SD 3.9, 30 females) from the population studied in Experiment 1, above completed the Autism-Spectrum Quotient (AQ) questionnaire consisting of 50 question items. The total AQ score was computed based on responses to all the 50 items. There were 10 questions for each of five subscales: social skills, attention switching, attention to detail, communication and imagination (see ref. S6 for full details of the questionnaire).

Supplemental Experimental Procedures

Experiment 1. Voxel-Based Morphometry of Loneliness

Participants

For the VBM experiment, a total of 108 healthy volunteers with normal or corrected to normal vision (aged 18-32, mean 23.5 ± 4.37 SD, 62 female) were recruited from the University College London subject pool. The experiments were approved by the local ethics committee and participants gave written informed consent.

Assessment of Loneliness

All participants were asked to fill out the UCLA Loneliness Scale Questionnaire [S8]. The questionnaire consists of 20 items that are derived from statements from lonely individuals to describe the feeling of loneliness [S9]. Participants were asked to indicate how often they feel the way described by each of the statements on a scale from 1 to 4, with 1 indicating "never" and 4 meaning "always". The statements include, for example, 'How often do you feel isolated from others?'.

MRI Data Acquisition

MR images were acquired on a 1.5-T Siemens Sonata MRI scanner (Siemens Medical, Erlangen, Germany). High-resolution anatomical images were acquired using a T1- weighted 3-D Modified Driven Equilibrium Fourier Transform (MDEFT) sequence (TR= 12.24 ms; TE = 3.56 ms; field of view = 256 x 256 mm; voxel size = 1 x 1 x 1 mm).

VBM Preprocessing and Analysis

To perform optimized voxel-based morphometry analysis [S10], T1-weighted MR images were first segmented for GM and WM using the segmentation tools in Statistical Parametric Mapping 8 (SPM8, http://www.fil.ion.ucl.ac.uk/spm). Subsequently, we performed Diffeomorphic Anatomical Registration Through Exponentiated Lie Algebra (DARTEL) in SPM8 for inter-subject registration of the GM images [S11]. To ensure that regional gray matter volume is maintained after the registration, the registered images were modulated by the Jacobian determinant of the flow fields computed by DARTEL. The registered images were smoothed with a Gaussian kernel of 12mm full-width-half-maximum (FWHM) and were then transformed to Montreal Neurological Institute (MNI) stereotactic space using affine and non-linear spatial normalisation implemented in SPM8.

A multiple regression analysis was performed on the smoothed grey matter images in SPM8 to determine regions in which grey matter density showed a correlation with the UCLA Loneliness Scale [S8]. The age, gender and total gray matter volume of individual brain were included in the design matrix as covariates of no interest and were thus regressed out. Clusters were initially identified as contiguous groups of voxels that exceeded an uncorrected threshold of voxel-wise $p < 0.001$. We then employed a threshold of $p(corr) < 0.05$ corrected for multiple comparisons across the whole brain volume at a cluster level using non-stationary correction [S12] to identify significantly correlated regions.

Experiment 2. Social Perception and Loneliness

Participants: We contacted the participants in Experiment 1 and asked them to take part in follow-up experiments. Selection of participants was purely based on their availability and was not based on their data collected in Experiment 1. For the social perception experiments, a total of 22 healthy volunteers with normal or corrected to normal vision (aged 19-30, mean 22.7 ± SD 3.9, 15 females) were recruited from the study population in experiment 1, above. The experiments were approved by the local ethics committee and participants gave written informed consent.

Abnormal Gaze Detection Task

The gaze perception task was administered to investigate the participants' abilities to perceive abnormal gaze of another person. In this task, participants were shown three faces simultaneously and asked to choose which one of the three faces showed an abnormal gaze. On each trial, participants were shown a fixation cross (2000ms), followed by the stimuli (4000ms), followed by a blank screen (3000ms). During stimulus presentation three of sixteen models were shown simultaneously. None of the models gazed directly at the camera. For two models in each trial, both eyes gazed in the same direction, but for one model, each eye gazed to a different location (i.e. strabismic gaze). Participants were asked to indicate the model with the abnormal gaze by pressing a corresponding key. Participant responses were recorded from the onset of the stimuli. Performance was measured using an efficiency score combining reaction time and accuracy (i.e. proportion of correct responses/mean reaction for correct responses). One hundred and forty trials were completed (preceded by four practice trials). These trials were split into four blocks of thirty-five trials and were randomized within blocks. In each block, upright or inverted faces were presented in isolation (i.e. two blocks of upright face trials and two blocks of inverted face trials using the same sixteen models in each block). Data from upright face trials and from inverted face trials were averaged for each participant, because those two measures were highly correlated ($R=0.91$, $p < 0.001$). As expected, the efficiency scores (accuracy/RT) for upright faces were significantly higher than for inverted faces (0.338 vs 0.288, $T(28)=5.78$, $p < 0.001$). All images were grayscale and edited to the same size using Adobe Photoshop.

Emotional Expression Discrimination Task

This task investigated participants' abilities to match another's facial expressions [S13]. Participants were shown a "sample" face (250ms) followed by a fixation cross (1000ms), and finally a "target" face (250ms). Participants were asked to indicate whether the target facial expression was the same or different to the sample facial expression. A total of 72 trials (split between 2 blocks) were completed, with 36 target-sample pairs involving the same emotion and 36 target-sample pairs involving different emotions. Thirty-six grayscale stimuli from the Ekman and Friesen facial affect set [S14] were used; six female models portrayed each of the six basic facial expressions of emotion: anger, disgust, fear, happiness, sadness or surprise. The hair and neck of stimuli were removed using Adobe Photoshop. Identity always changed between sample-target pairs and each expression was presented an equal number of times.

Identity Discrimination Task

In the identity discrimination task, the same stimuli and procedure were used as the emotion discrimination task. Participants were, however, asked to indicate whether the sample and target face were the same or a different person. Half of the trials showed pairs with the same

identity and half with a different identity. Expression always changed between the sample and target face, and the six models were presented an equal number of times.

Films Emotion Recognition Task

This task investigated participants' abilities to recognize the emotional expressions of others [S15,S16]. Participants were presented with an adjective describing an emotional state followed by three images (each image shown for 500ms) of the same actor or actress displaying different facial expressions. Participants were asked to indicate which of the three images best portrayed the target emotional adjective. Three practice trials were followed by 58 test trials, which were split over two blocks of twenty-nine trials.

Analysis

Efficiency scores were computed as the ratio of accuracy divided by mean reaction time. The efficiency scores were also compared against the gray matter volume extracted from the pSTS cluster identified in Experiment 1. We computed the Pearson correlation between loneliness score and performance efficiency score for each of the tasks. While we did not control for age and sex in our report, all the significant results reported in the behavioural studies (Experiments 2, 3, 4 and 5) remained significant even after controlling for demographic variables (age and sex).

Experiment 3. Social Network Size and Loneliness

Social Network Size Questionnaire

Forty-five participants were recruited from UCL student community (aged 18-30 mean 23.2 ± SD 3.6, 52 females). These participants were a subset of the participants who participated in our previous study on brain structure correlates of online social network size [S7]. Written informed consent was obtained and the study was approved by the local ethics committee. The questionnaire was adapted from ref. [S17]. It consisted of the following nine questions.

1. How many were present at your 18th or 21st Birthday Party?
2. If you were going to have a party now, how many people would you invite?
3. What is the total number of friends in your phonebook?
4. Write down the names of the people that you would send a text message to marking a celebratory event (e.g. Birthday, Christmas, new job, good exam result etc.). How many people is that?
5. Write down the names of people in your phonebook you would meet for a chat in a small group (1-3 people). How many people is that?
6. How many friends have you kept from school and university that you could have a friendly conversation with now?
7. How many friends do you have on "Facebook"?
8. How many friends do have from outside school or university?
9. Write down the names of the people you feel you could ask a favour of and expect to have it granted. How many people is that?

These questions loaded strongly onto a single factor [S7,S17]. We computed a normalized social network size for each participant by averaging the z-scores for the questions items.

Experiment 4. Anxiety and Loneliness

State-Trait Anxiety Inventory (STAI)

Sixty-one participants (aged 18-39, mean 23.5 ± SD 4.5, 43 females) from the population studied in Experiment 1 completed the STAI for trait anxiety consisting of 20 question items (Form Y) [S18].

Experiment 5. Empathy and Loneliness

Interpersonal Reactivity Index

Ninety-five participants (aged 18-39, mean 22.3 ± SD 4.3, 53 females) from the population studied in Experiment 1 completed the Interpersonal Reactivity Index (IRI) questionnaire consisting of 28 question items [S19]. There were four subscales; fantasy scale (FS), perspective taking (PT), personal distress (PD) and empathic concern (EC) (see ref. S19 for full details of the questionnaire). Each subscale contained seven items. They were measured on a five point Likert scale ranging from 0 (“Does not describe me well”) to 4 (“Describes me very well”). FS measures the tendency of an individual to transpose themselves into fictional situations. PT measures the tendency to think from another person’s perspective. PD measures the tendency to feel negative emotion when observing others undergoing affectively negative situations. EC measures the tendency to feel compassion and sympathy for other individuals.

Supplemental References

S1. Mundy, P., Sigman, M., Ungerer, J. and Sherman, T. (1986). Defining the social deficits of autism: the contribution of non-verbal communication measures. J. Child. Psychol. Psychiatry *27*, 657-669.

S2. Baron-Cohen, S., Campbell, R., Karmiloff-Smith, A., Grant, J. and Walker, J. (1999). Are children with autism blind to the mentalistic significance of the eyes? Br. J. Dev. Psychol. *13*, 379-398.

S3. Frith, C.D. and Frith, U. (1999). Interacting minds – a biological basis. Science *286*, 1692.

S4. Bauminger, N. and Kasari, C. (2000). Loneliness and friendship in high-functioning children with autism. Child Dev. *71*, 447-456.

S5. Bauminger, N., Shulman, C. and Agam, G. (2003). Peer interaction and loneliness in high-functioning children with autism. J. Autism Dev. Disord. *33*, 489-507.

S6. Baron-Cohen, S., Wheelwright, S., Skinner, R., Martin, J. and Clubley, E. (2001). The Autism Spectrum Quotient (AQ): Evidence from Asperger Syndrome/high-functioning autism, males and females, scientists and mathematicians. J. Autism Dev. Disord. *31*, 5–17.

S7. Kanai, R., Bahrami, B., Roylance, R. and Rees, G. (2012). Online social network size predicts human brain structure. Proc. R. Soc. B. *279*, 1327-1334.

S8. Russell, D. (1996). The UCLA Loneliness Scale (version 3): Reliability, validity and factor structure. J. Pers. Assess. *66*, 20- 44.

S9. Russell, D., Peplau, L.A. and Ferguson, M.L. (1978). Developing a measure of loneliness. J. Pers. Assess. *42*, 290-294.

S10. Ashburner, J. and Friston, K.J. (2000). Voxel-based morphometry – the methods. NeuroImage *11*, 805-821.

S11. Ashburner, J. (2007). A fast diffeomorphic image registration algorithm. NeuroImage *15*, 95-113.

S12. Hayasaka, S., Phan, K.L., Liberzon, I., Worsley, K.J. and Nichols, T.E. (2004). Nonstationary cluster-size inference with random field and permutation methods. NeuroImage *22*, 676-687.

S13. Pitcher, D., Garrido, L., Walsh, V. and Duchaine, B.C. (2008). Transcranial magnetic stimulation disrupts the perception and embodiment of facial expressions. J. Neurosci*. 28*, 8929-8933.

S14. Eckman, P. and Friesen, W. (1976). Pictures of facial affect. (Consulting Psychologists Press, Palo Alto, CA).

S15. Garrido, L., Furl, N., Dranganski, B., Weiskopf, N., Stevens, et al. (2009). Voxel-based morphometry reveals reduced grey matter volume in the temporal cortex of developmental prosopagnosics. Brain *132*, 3443-3455.

S16. Banissy, M.J., Garrido, L., Kusnir, F., Duchaine, B., Walsh, V. and Ward, J. (2011). Superior facial expression, but not identity recognition, in mirror-touch synesthesia. J. Neurosci. *31*, 1820-1824.

S17. Stileman, E. and Bates, T. (2007). Construction of the Social Network Score Questionnaire for undergraduate students, and an examination of the pre-requisites for large social networks in humans. Edinburgh Research Archive.

S18. Spielberger, C.D. (1983). Manual for the State-Trait Anxiety Inventory (STAI). (Consulting Psychologists Press, Palo Alto, CA).

S19. Davis, M.H. (1983). Measuring individual differences in empathy: evidence for a multidimensional approach. J. Pers. Soc. Psychol. *44*, 113-126.
